# Pan‐Cancer Single‐Cell and Spatial‐Resolved Profiling Reveals the Immunosuppressive Role of APOE+ Macrophages in Immune Checkpoint Inhibitor Therapy

**DOI:** 10.1002/advs.202401061

**Published:** 2024-04-03

**Authors:** Chuan Liu, Jindong Xie, Bo Lin, Weihong Tian, Yifan Wu, Shan Xin, Libing Hong, Xin Li, Lulu Liu, Yuzhi Jin, Hailin Tang, Xinpei Deng, Yutian Zou, Shaoquan Zheng, Weijia Fang, Jinlin Cheng, Xiaomeng Dai, Xuanwen Bao, Peng Zhao

**Affiliations:** ^1^ Department of Medical Oncology The First Affiliated Hospital School of Medicine Zhejiang University Hangzhou 310003 China; ^2^ State Key Laboratory of Oncology in South China Guangdong Provincial Clinical Research Center for Cancer Sun Yat‐sen University Cancer Center Guangzhou 510060 China; ^3^ College of Computer Science and Technology Zhejiang University Hangzhou 310053 China; ^4^ Innovation Centre for Information Binjiang Institute of Zhejiang University Hangzhou 310053 China; ^5^ Changzhou Third People's Hospital Changzhou Medical Center Nanjing Medical University Changzhou 213000 China; ^6^ School of software Zhejiang University Ningbo 315100 China; ^7^ Department of Genetics Yale School of medicine New Haven CT 06510 USA; ^8^ Department Chronic Inflammation and Cancer German Cancer Research Center (DKFZ) 69120 Heidelberg Germany; ^9^ Breast Disease Center The First Affiliated Hospital Sun Yat‐Sen University Guangzhou 510060 China; ^10^ State Key Laboratory for Diagnosis and Treatment of Infectious Diseases National Clinical Research Center for Infectious Diseases National Medical Center for Infectious Diseases Collaborative Innovation Center for Diagnosis and Treatment of Infectious Diseases The First Affiliated Hospital Zhejiang University School of Medicine Zhejiang University Hangzhou 310003 China

**Keywords:** APOE^+^ macrophages, immune checkpoint inhibitor, machine learning algorithm, pan‐cancer, single‐cell RNA sequencing

## Abstract

The heterogeneity of macrophages influences the response to immune checkpoint inhibitor (ICI) therapy. However, few studies explore the impact of APOE^+^ macrophages on ICI therapy using single‐cell RNA sequencing (scRNA‐seq) and machine learning methods. The scRNA‐seq and bulk RNA‐seq data are Integrated to construct an M.Sig model for predicting ICI response based on the distinct molecular signatures of macrophage and machine learning algorithms. Comprehensive single‐cell analysis as well as in vivo and in vitro experiments are applied to explore the potential mechanisms of the APOE^+^ macrophage in affecting ICI response. The M.Sig model shows clear advantages in predicting the efficacy and prognosis of ICI therapy in pan‐cancer patients. The proportion of APOE^+^ macrophages is higher in ICI non‐responders of triple‐negative breast cancer compared with responders, and the interaction and longer distance between APOE^+^ macrophages and CD8^+^ exhausted T (Tex) cells affecting ICI response is confirmed by multiplex immunohistochemistry. In a 4T1 tumor‐bearing mice model, the APOE inhibitor combined with ICI treatment shows the best efficacy. The M.Sig model using real‐world immunotherapy data accurately predicts the ICI response of pan‐cancer, which may be associated with the interaction between APOE^+^ macrophages and CD8^+^ Tex cells.

## Graphical abstract

The M.Sig model is constructed by pan‐cancer scRNA‐seq and bulk RNA‐seq from ICI cohorts with multiple machine learning algorithms. The potential mechanism in TNBC by which the M.Sig model can predict ICI response is the interaction between APOE^+^ macrophages and CD8^+^ Tex cells.

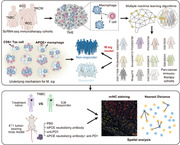

## 1 Introduction

Immune checkpoint inhibitors (ICIs) are increasingly becoming a mainstay of cancer treatment strategies. Prior research has demonstrated the efficacy in treating a variety of cancers, including lung cancer, melanoma,^[^ ^1^ ^]^ and gastrointestinal cancer.^[^ ^2^ ^]^ However, in addition to the occurrence of some clinical side effects,^[^ ^3^ ^]^ the limited response rate is a significant hurdle that impedes widespread clinical application of immunotherapy. This has encouraged the development of biomarker studies to predict response to immunotherapy, with optimization of treatment combinations to combat immunological resistance. Traditional biomarker studies largely focus on whole‐exome sequencing (WES) or RNA sequencing (RNA‐seq) of tumor tissues, and these approaches only reflect the average genetic profile of tumors,^[^ ^4^ ^]^ such as expression of programmed cell death‐ligand 1 (PD‐L1),^[^ ^5^ ^]^ microsatellite instability (MSI)^[^ ^6^ ^]^ and the tumor mutation burden (TMB).^[^ ^5^ ^]^ With the advent of single‐cell RNA sequencing (scRNA‐seq), there are a variety of techniques for analyzing gene expression at the cellular level, facilitating the identification of more sensitive markers.

The tumor microenvironment (TME) contains CD4^+^ T cells and CD8^+^ T cells, which predominantly act as effector cells in ICI therapy. Their degree of infiltration can be used as a reliable predictor for response to ICIs.^[^ ^7^ ^]^ However, the majority of patients have an immune‐exclusion phenotype, and according to recent research, myeloid infiltration (macrophages, monocytes, and granulocytes) plays a key mediator function in this phenomenon.^[^ ^8^ ^]^ Among these cells, a series of subpopulations of macrophages separated by scRNA‐seq have been found to exert different immune functions, such as SPP1^+^ macrophages,^[^ ^9^ ^]^ APOE^+^ macrophages,^[^ ^10^ ^]^ and tissue‐resident macrophages (TRMs).^[^ ^11^ ^]^ Most subsets can be reprogrammed to up‐regulate immune checkpoints and various inflammatory chemokines, thereby inhibiting antitumor immunity, such as FABP4^+^ macrophages,^[^ ^12^ ^]^ MRC1^+^ CCL18^+^ macrophages^[^ ^13^ ^]^ and IL4I1^+^ CD274^+^ IDO1^+^ macrophages.^[^ ^14^ ^]^ Nevertheless, some macrophage subsets, such as CD68^+^ macrophages, have a protective role in the prognosis of cancer patients.^[^ ^15^ ^]^ The main reasons for this heterogeneity are transcriptomic diversity and the distinct pathway activities.^[^ ^16^ ^]^ In addition, transcriptomic analysis suggested that there may be an interaction between macrophages and T cells.^[^ ^17^ ^]^ Li et al. found that CD8^+^ T cells in tumors would be presented with antigens by tumor‐associated macrophages (TAMs) expressing IRF8 to promote T cell depletion.^[^ ^18^ ^]^ Previous studies have mainly explored the relationship between macrophage‐related genes, the TME and immunotherapy effects,^[^ ^19^ ^]^ whereas few have combined pan‐cancer scRNA‐seq with machine learning algorithms to explore the correlation between macrophage subpopulations and T cells that effecting immunotherapy response.

In this study, integrative scRNA‐seq and bulk transcriptome data were utilized to build an M.Sig model using eight machine learning algorithms, and the model showed efficacy in predicting response to ICI‐based immunotherapy of pan‐cancer. In triple negative breast cancer (TNBC), the potential mechanism affecting the efficacy of immunotherapy is the interaction between APOE^+^ macrophages and CD8^+^ exhausted T (Tex) cells, which was verified by in vivo experiments and multiplex immunohistochemistry (mIHC) staining.

## 2 Results

### 2.1 Immune Landscape in Multiple Cancers with ICI Treatments

**Figure 1:**
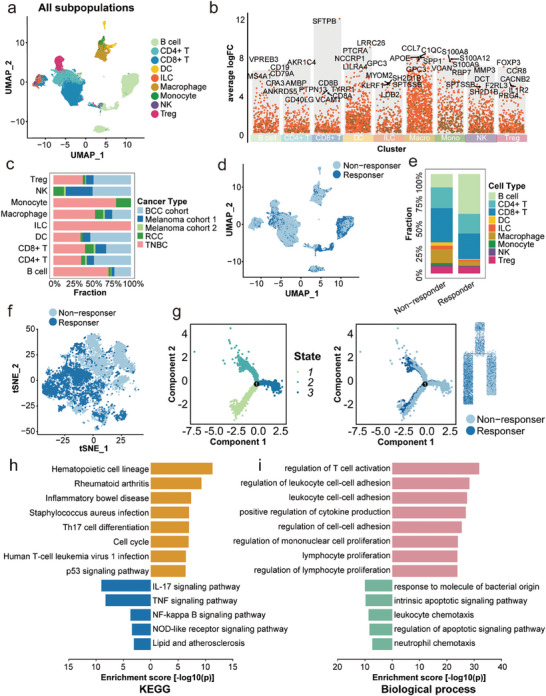
Immune cell landscape of ICI‐treated pan‐cancers. a) Color‐coded UMAP plot of cell types in five cohorts with ICI treatment. b) Feature genes of each cell type. c) The proportion of cohorts in each cell type. d) The distribution of ICI responders and non‐responders in all cells. e) The proportion of each cell type between responders and non‐responders. f) The distribution of ICI responders and non‐responders in macrophages. g) Trajectory analysis of ICI responders and non‐responders. h) KEGG analysis of DEGs between ICI responders and non‐responders. i) Biological Process of GO enrichment of DEGs between immunotherapy responders and non‐responders. ICI: immune checkpoint inhibitor; UMAP: uniform manifold approximation and projection; KEGG: Kyoto Encyclopedia of Genes and Genomes; DEGs: differential expressed genes; GO: Gene Ontology.

Five scRNA‐seq cohorts were used to explore the relationship between immune cell subsets and immunotherapy response, in which melanoma was the most prevalent (n = 35), followed by basal cell carcinoma (BCC) (n = 10) and TNBC (n = 9) (Figure S1a, Supporting Information). In terms of ICI response, there were significantly fewer responders than non‐responders, except for renal cell carcinoma (RCC) (Figure S1c, Supporting Information). To identify distinct types of cell clusters, we first used uniform manifold approximation and projection (UMAP) to lower the dimension and cluster the cells of five scRNA‐seq cohorts with ICI treatment involving four kinds of cancer (**Figure** **1**a). In general, nine cell types were recognized. Expression of CD8A, CD8B, and GZMK was considerably higher in CD8^+^ T cells, and FOXP3, TNFRSF4, and TNFRSF18 were more highly expressed in regulatory T cells (Tregs). Additionally, macrophages were marked by APOE, APOC1, C1QC, and C1QB expression (Figure 1b). Of these cells, TNBC, BCC, and melanoma comprised the largest proportion; only B cells, CD4^+^ T cells, CD8^+^ T cells, Tregs, and macrophages were shared by the five cohorts (Figure 1c), showing that they are typically present in tumor tissues. And TNBC had the highest concentration of macrophages, followed by BCC (Figure S1b, Supporting Information). To comprehend the heterogeneity of the TME between responders and non‐responders, we analyzed the ratio of cell types. As depicted in Figure 1d,e, the proportion of macrophages was significantly lower but the proportion of B cells higher in responders, indicating that these cell types may influence ICI response in patients.

Then we conducted differential gene analysis at the level of cell type between responders and non‐responders. It shows that there are most differentially expressed genes (DEGs) in macrophages, regardless of whether the patients were responders or non‐responders, confirming their crucial involvement in immunotherapy (Figure S1d,e, Supporting Information). To gain a deeper understanding of the effect macrophages have on immunotherapy, we further reduced the dimensions of macrophages with T‐distributed stochastic neighbor embedding (tSNE). Figure 1f shows that macrophages could be subdivided into different clusters, and responders and non‐responders are also clearly separated, demonstrating the existence of macrophage subset characteristics that influence immunotherapy. Trajectory analysis showed that macrophages can differentiate into two groups of subtypes as the tumor progresses, as responders and non‐responders can be identified within these two groups, suggesting that macrophages can differentiate between non‐responders and responders (Figure 1g).

To explore the distinction between responders and non‐responders, we evaluated expression profiles in macrophages and discovered that various genes, including CCL2, APOE, and TIMP1, had different expression levels (Figure S1f, Supporting Information). Previous research has supported the role of these genes as cancer prognosis‐related molecules or tumor immune regulators.^[^ ^20^ ^]^ Gene Ontology (GO) and Kyoto Encyclopedia of Genes and Genomes (KEGG) functional enrichment analyses were conducted on DEGs of macrophages. The differential genes were enriched in a number of immune‐related pathways, such as the TH17 signaling pathway, TNF signaling pathway, and antigen processing and presentation (Figure 1h). Similarly, they are mostly involved in the generation and regulation of cytokines, as well as the adhesion and proliferation of leukocytes, according to biological process of GO enrichment (Figure 1i).

### 2.2 The scRNA‐seq‐Inspired M.Sig Model Predicts ICI Response and Prognosis

**Figure 2:**
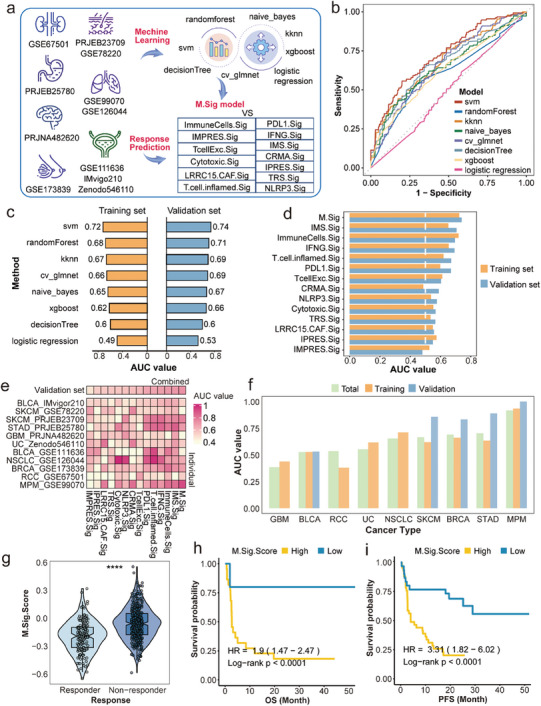
Construction of the M.Sig model to predict ICI response and prognosis. a) Workflow of the construction and validation of the M.sig model. b) The AUC of eight machine learning algorithms in the training set. c) Eight AUC values of the training set and the test set of eight machine learning algorithms. d) Comparison of the AUCs among the M.Sig model and other thirteen immune signatures in the training set and the test set. e) Comparison of the AUCs in each cohort. f) The AUCs of the M.Sig model in each cancer type. g) Different M.Sig.Score between ICI responders and non‐responders in the training set. h,i) OS and PFS of high or low M.Sig.Score in the training set. ICI: immune checkpoint inhibitor; AUC: area under the curve; OS: overall survival; PFS: progression‐free survival.

The aforementioned results indicated that macrophages alter the immune response, and the DEGs in Figure S1f (Supporting Information) were further examined. We obtained eleven ICI cohorts including various solid tumors, and gathered corresponding RNA‐seq data and clinical data to better investigate the predictive ability of these DEGs. In total, eight machine learning algorithms were used to optimize the model with the greatest significance, which was compared with the immune‐related signature from previous studies (**Figure** **2**a). The eleven datasets contained nine solid tumor types, with bladder cancer (BLCA) accounting for the most cases (n = 359), followed by melanoma (n = 99) and breast cancer (BRCA) (N = 71) (Figure S2a, Supporting Information). However, for most malignancies, responders did not exceed 50%, suggesting the limit of immunotherapy efficacy (Figure S2b, Supporting Information). First, the eight machine learning algorithms were utilized to train models, and then repeated cross‐validation (CV) was used to refine the parameters of each model. We harvested eight models after training. Among the respective area under the curves (AUCs), the AUC of 0.72 for support vector machine (svm) model was the greatest, which was chosen as the model (Figure 2b). When using the validation set to verify each model, we found that the AUC of the svm model remained the highest, at 0.74 (Figure 2c), indicating that the model we developed was stable and effective. The AUC was then computed utilizing feature ranking, and the AUC values of both the training set and the verification set were comparable to those before when 35 features were included (Figure S2c, Supporting Information), such as CXCL10, MAP1A, and CCL8, which was defined as M.Sig model (Figure S2d, Supporting Information).

Predicting the effect of immunotherapy is conducive to improving the accuracy of treatment. As a result, prior research has also generated a series of prediction models. We compiled and compared thirteen immune‐related signatures with the M.Sig model. In both training and verification sets, the AUC of the M.Sig model was greater than the others (Figure 2d). Then, we compared the AUC of each signature at the level of an individual dataset and found that most signatures were only effective for one or two cohorts (Figure 2e). In GSE120644, for instance, the AUCs of both NLRP3.Sig and Cytotoxic.Sig surpassed 0.8, whereas in other datasets it was lower, at approximately 0.6. In contrast, the M.Sig model performed well in most cohorts. In addition, the AUC exceed 0.75 for malignant pleural mesothelioma (MPM), stomach adenocarcinoma (STAD), skin cutaneous melanoma (SKCM), and BRCA (Figure 2f), even though AUCs could not be calculated in the test set of some cancers due to the small number of patients (non‐small cell lung cancer [NSCLC], urothelial carcinoma [UC] and RCC). This highlights the model's ability to forecast ICI effectiveness across cancers. On the basis of building the machine learning model, the expression of non‐responder up‐regulated genes in the M.Sig model was subtracted from that of down‐regulated genes to derive M.Sig.Score. In the training set, the score for non‐responders was greater than that for responders (*P* < 0. 0001) (Figure 2g), and the overall survival (OS) and progression‐free survival (PFS) of patients in the low‐score group were better than those in the high score group (all *P* < 0.0001) (Figure 2h,i). This was the same as the result for the verification set (Figure S2e‐g, Supporting Information), demonstrating that M.Sig.Score is related to nonresponse to ICIs and poor prognosis. Taken together, we developed an M.Sig model to predict ICI response, and the effect was better than that of other recognized models.

### 2.3 Molecular Characteristics of the M.Sig Model in Pan‐Cancer from TCGA

We collected data for thirty kinds of solid tumors from the database The Cancer Genome Atlas (TCGA) and used these data to confirm our findings. First, we performed a comprehensive analysis of the M.Sig model and 75 immune‐related genes and found that the model was generally inversely linked to the expression level of immune‐related genes (Figure S3a, Supporting Information). Then, we assessed the status of immune cell infiltration to further describe the TME. Compared to monocytes and myeloid cells, cancers with high M.Sig.Score showed fewer cytotoxic immune cells, including CD8^+^ T cells and cytotoxic lymphocytes (Figure S3b, Supporting Information). In conclusion, these findings reveal a negative correlation between the M.Sig model and antitumor immunity. As the TMB and MSI status are significant immunotherapy biomarkers, we examined their interactions with the M.Sig model. As illustrated in Figure S3c (Supporting Information), we observed a negative association between the M.Sig model and TMB (R = −0.6, *P* < 0.001). Similarly, MSI‐High (MSI‐H) shows a lower M.Sig.Score than MSI‐Low (MSI‐L) and microsatellite stable (MSS) (*P* < 0.001) (Figure S3d, Supporting Information). The negative connection between the M.Sig model and TMB and MSI‐H further demonstrate that the M.Sig model may predict the therapeutic effect.

### 2.4 The Proportion of APOE^+^ Macrophages was Higher in ICI‐Treated TNBC Non‐Responders

**Figure 3:**
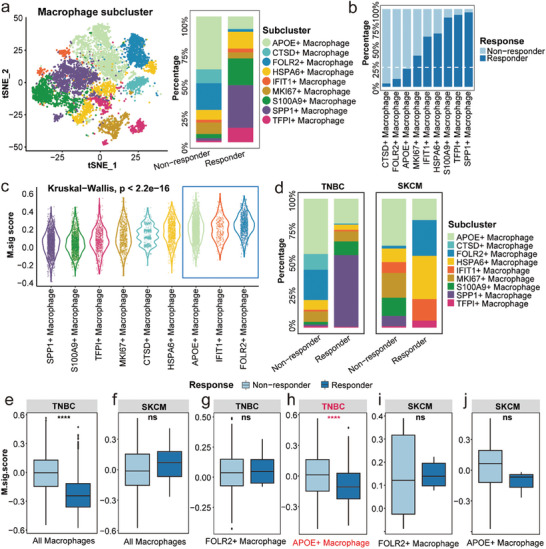
Different M.Sig.Score in macrophage subpopulations between responders and non‐responders. a) Colour‐coded tSNE plot of eleven macrophage subgroups and the proportion of subgroups in ICI responders and non‐responders. b) The proportions of each macrophage subgroup in ICI responders and non‐responders. c) M.Sig.Score of each macrophage subset. d) The proportion of each macrophage subgroup in ICI responders and non‐responders of TNBC and SKCM. e–j) The M.Sig.Score of macrophage subsets between ICI responders and non‐responders of TNBC and SKCM. tSNE: t‐distributed stochastic neighbor embedding; ICI: immune checkpoint inhibitor; TNBC: triple negative breast cancer; SKCM: skin cutaneous melanoma.

After the construction of the M.Sig model, it was necessary to further understand its biological significance based on scRNA‐seq. Using tSNE, we observed that macrophages could be classified into eleven subgroups, and the proportion of these cells was significantly different between responders and non‐responders (**Figure** **3**a). For instance, the proportions of APOE^+^ macrophages, CTSB^+^ macrophages, and FOLR2^+^ macrophages were much higher in non‐responders, whereas the proportions of S100A9^+^ macrophages, SPP1^+^ macrophages, and TFPI^+^ macrophages were lower, indicating that these cells may play a crucial role in the TME. The proportion of most cell types was high in TNBC, such as APOE^+^ macrophage and CTSD^+^ macrophage (Figure S4a, Supporting Information). Besides, we discovered the largest number and percentage of APOE^+^ macrophages, FOLR2^+^ macrophages, and CTSD^+^ macrophages among non‐responders (Figure 3b; Figure S4b, Supporting Information). To further understand the relationship between the presence of each subgroup and immune function, we compared immune evasion and immune checkpoint genes, immune‐related pathways and transcription factor (TF) activities of each subcluster. Among immune‐related genes, LAG3, PPARG, and CD274 showed a low expression ratio across all categories. Specifically, VSIG4 and CSF1R levels were elevated in FOLR2^+^ macrophages (Figure S4d, Supporting Information). Previous research has shown that macrophage‐related pathways, including antigen presentation, M1 regulation, and proteasome, are associated with macrophage activity, illustrating distinctions between distinct subgroups (Figure S4e, Supporting Information). In FOLR2^+^ macrophages and CTSB^+^ macrophages, the M1 cultured, proteasome, IFN‐*γ* response, and IFY‐stimmd MDM function were diminished compared to those of other macrophages (Figure S4e, Supporting Information). The TF activities of SPP1^+^ macrophages and CTSB^+^ macrophages were also considerably different (Figure S4c, Supporting Information). TBX21, for instance, can mediate T‐cell activity and stimulate antitumor immunity,^[^ ^21^ ^]^ and its expression in CTSB^+^ macrophages and SPP1^+^ macrophages was much lower than that in other subtypes. In contrast, CCL5^+^ macrophages and TFPI^+^ macrophages exhibited a considerably greater level of expression. Intriguingly, M.Sig.Score of these three subgroups were also among the top three (*P* < 0.0001) (Figure 3c). These findings indicated that a rise in the number of these three types of cells is not favorable for ICI therapy efficacy. Expression of the 35 genes in the M.Sig model was then examined (Figure S4f, Supporting Information), and the expression levels of CFI and CCL8 were significantly greater in these three subpopulations than in others.

In these cancer types, TNBC and SKCM contain all the macrophage subsets above, so we compared the proportion of macrophage subsets of responders and non‐responders in TNBC and SKCM. The results showed that FLOR2^+^ macrophage proportion was higher in responders of SKCM, while this of S100A9^+^ macrophages was lower, which was inconsistent with the results of all cancer and TNBC, suggesting heterogeneity of cancers (Figure 3d). Then, we compared M.Sig.Score of all macrophages and top two subsets (FLOR2^+^ macrophages and APOE^+^ macrophages) that may have a negative impact on immunotherapy in TNBC and SKCM. In all macrophages and APOE^+^ macrophages of TNBC patients, M.Sig.Score are higher in non‐responders, but the result is opposite in SKCM with no significant difference (Figure 3e‐j). Therefore, we speculate that APOE^+^ macrophage is an important factor in the failure of ICI therapy in TNBC patients.

### 2.5 APOE^+^ Macrophages Interact with CD8^+^ Tex Cells to Affect ICI Response in TNBC

**Figure 4:**
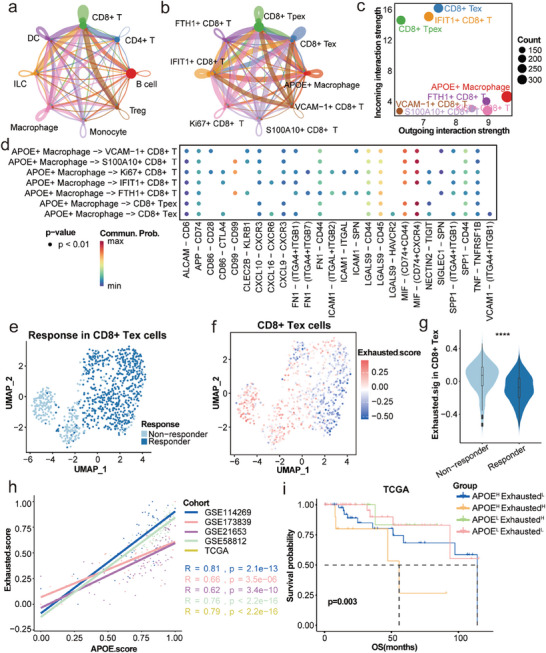
The interaction between APOE^+^ macrophage and CD8^+^ Tex cells affects ICI response in TNBC. a) Cell‐cell interactions among immune cell types. b) Cell–cell interactions among APOE^+^ macrophages and different CD8^+^ T cells. c) The relationship between differential outgoing interactions and incoming interaction strength for CD8^+^ T cells and APOE^+^ macrophages. d) Immune‐related ligands and receptors for signal communication between APOE^+^ macrophages and distinct CD8^+^ T cell subsets. e) Color‐coded UMAP plot of responders and non‐responders in CD8^+^ Tex cells. f) Exhausted score of CD8^+^ Tex cells in UMAP. g) The Exhausted.score of CD8^+^ Tex cells between ICI responders and non‐responders. h) Correlation of Exhausted.score with APOE.score in TNBC from GEO and TCGA cohorts. i) OS analysis of distinct Exhausted.score and APOE.score in TNBC from TCGA cohort. ICI: immune checkpoint inhibitor; UMAP: uniform Manifold Approximation and Projection; TNBC: triple negative breast cancer; GEO: Gene Expression Omnibus; TCGA: The Cancer Genome Atlas; OS: overall survival.

In order to further determine the mechanism of APOE^+^ macrophages influencing the efficacy of immunotherapy in TNBC patients, we explored interactions among the nine cell types by investigating distinct intercellular communication in TNBC. It was demonstrated that macrophages and CD8^+^ T cells engage in most cellular communication (**Figure** **4**a). In responders and non‐responders, CD8^+^ T cells exhibited increased incoming communication intensity, whereas macrophages exhibited increased outgoing communication intensity (Figure S5a, Supporting Information). We performed dimensionality reduction clustering on T cells and identified a total of eleven subtypes (Figure S5b, Supporting Information). Then, cellular communication analysis was conducted between CD8^+^ T cells and APOE^+^ macrophages. APOE^+^ macrophages and CD8^+^ Tex cells engage in most cellular communication (Figure 4b), and CD8^+^ Tex cells exhibited increased incoming communication intensity, whereas APOE^+^ macrophages exhibited increased outgoing communication intensity (Figure 4c). Additionally, the interaction of MIF‐(CD74+CXCR4) on APOE^+^ macrophages was strong (Figure 4d), which may influence ICI efficacy via immunosuppressive interaction with CD8^+^ Tex cells and CD8^+^ progenitor exhausted T (Tpex) cells. In CD8^+^ Tex cells, there are some DEGs between responders and non‐responders (Figure S5c, Supporting Information), such as GZMK and MGP.

To confirm the role of CD8^+^ Tex cells in ICI response, uniform Manifold Approximation and Projection (UMAP) was used to display the distribution of them in TNBC, and calculated the corresponding Exhausted.score (Figure 4e,f). Compared to non‐responders, responders had a lower Exhausted.score in CD8^+^ Tex cells (Figure 4g). Then, we also showed the Exhausted.score of CD8^+^ Tpex cells between responders and non‐responders, which was higher in non‐responders (Figure S5d‐f, Supporting Information). These results indicated that an increase of CD8^+^ Tex cells leads to poorer immunotherapy efficacy, while CD8^+^ Tpex cells is the opposite. Based on the above results, we speculate that the response to ICIs is related to the interaction of APOE^+^ macrophages with CD8^+^ Tex. The transcriptional data of TNBC was obtained from TCGA and Gene Expression Omnibus (GEO), we calculated the corresponding Exhausted.score and APOE.score, and found that there was a significant positive correlation between them (Figure 4h). Then we divided TNBC patients from the TCGA database into two groups based on the APOE.score, and patients with high APOE.score have significantly shorter OS (*P* = 0.048) (Figure S5g, Supporting Information). After incorporating the Exhausted score as the grouping criterion, patients with a high APOE score and high Exhausted score had the worst prognosis (*P* = 0.003) (Figure 4i), indicating that APOE^+^ macrophages may interact with CD8^+^ Tex cells to affect the efficacy of ICIs and the prognosis of TNBC patients.

### 2.6 The Distance Between APOE^+^ Macrophages and CD8^+^ Tex Cells was Verified by Clinical TNBC Samples

**Figure 5:**
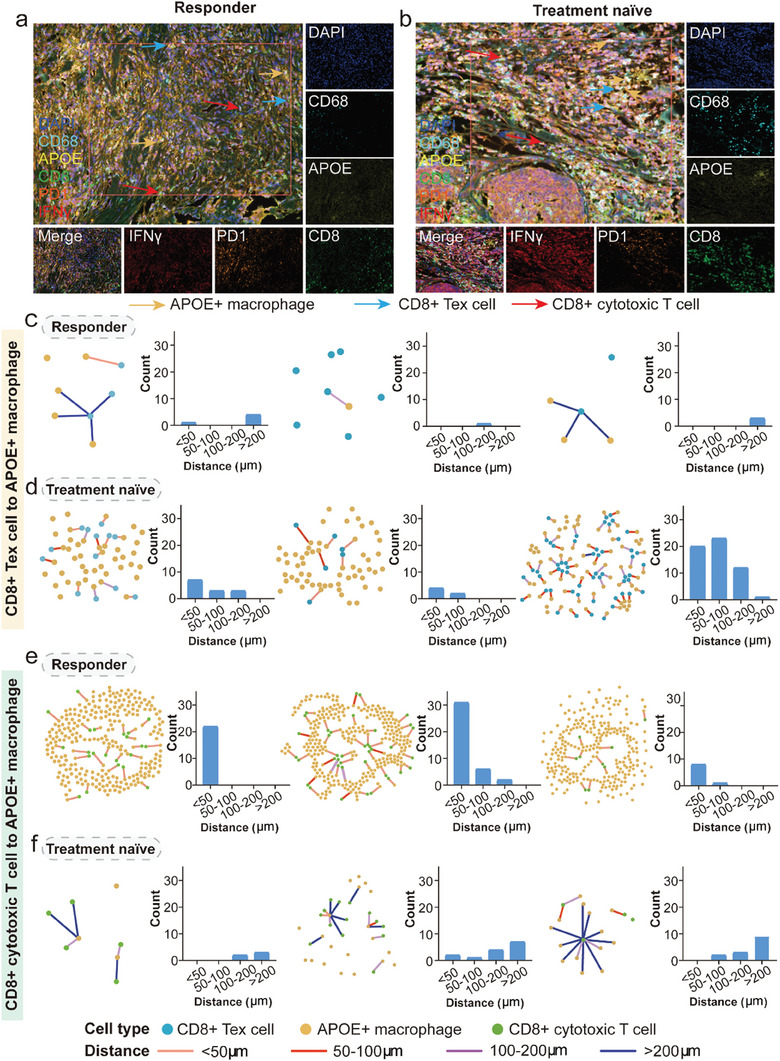
Spatial mIHC staining distinguishes the distance between APOE^+^ macrophages and CD8^+^ Tex cells and CD8^+^ cytotoxic T cells in human TNBC tissues. a,b) The mIHC images of indicated APOE^+^ macrophages (orange arrows), CD8^+^ Tex cells (blue arrows), and CD8^+^ cytotoxic T cells (red arrows) in ICI responder (left) or TN (right) TNBC tumor sections. c,d) The distances and their measurement between APOE^+^ macrophages and CD8^+^ Tex cells in ICI responder and TN patients. e,f) The distances and its measurement between APOE^+^ macrophages and CD8^+^ cytotoxic T cells in ICI responder and TN patients. mIHC: multiplex immunohistochemistry; ICI: immune checkpoint inhibitor; TN: treatment naïve; TNBC: triple negative breast cancer.

Clinical TNBC samples of ICI‐responders and treatment naïve patients were selected for mIHC staining. Compared with treatment naïve (TN) patients, the distances between APOE^+^ macrophages and CD8^+^ Tex cells were longer in responders, while shorter between APOE^+^ macrophages and CD8^+^ cytotoxic T cells (**Figure** **5**a,b). To further specify these results, we conducted additional spatial analyses on the mIHC images, measuring the nearest distance between CD8^+^ Tex cells and CD8^+^ cytotoxic T cells to APOE^+^ macrophages. Among the responders, the majority of CD8^+^ Tex cells were situated more than 200 µm away from APOE^+^ macrophages (Figure 5c), whereas in TN patients, the number of CD8^+^ Tex cells was greater and their proximity was closer (Figure 5d). However, when measuring the distance between CD8^+^ cytotoxic T cells and APOE^+^ macrophages, the findings were reversed (Figure 5e,f), indicating that the interaction between CD8^+^ Tex cells and APOE^+^ macrophages is one of the factors contributing to the failure of ICI therapy.

### 2.7 APOE Inhibitors can Enhance the Efficacy of ICI Therapy

**Figure 6:**
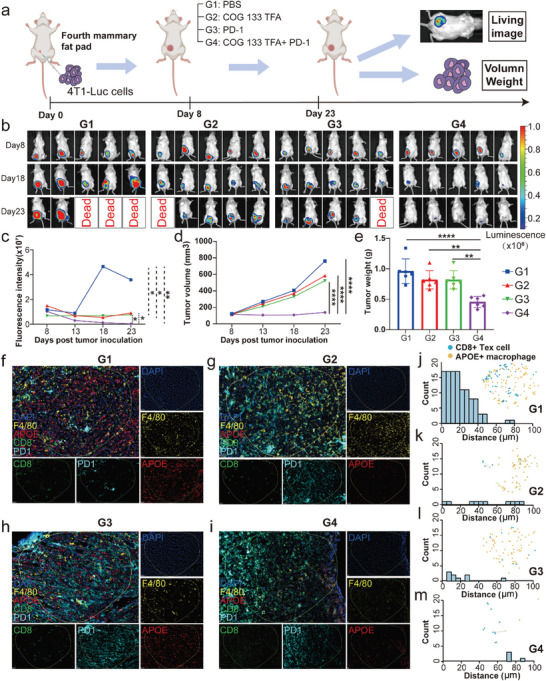
APOE inhibitors can enhance the efficacy of ICI therapy. a) In vivo experimental procedure in mice. b) Results of live imaging of G1–G4 mice. c) Comparison of fluorescence intensity in live imaging of G1–G4 mice. d) Changes in tumor volume of G1–G4 mice after treatment. e) Comparison of tumor weight in G1–G4 mice after four times treatment. f–i) Spatial mIHC staining of G1–G4 mice breast cancer sections (DAPI, F4/80, APOE, CD8, and PD1). j–m) The distance of APOE^+^ macrophages and CD8^+^ Tex cells in G1‐G4 mice. ICI: immune checkpoint inhibitor; mIHC: multiplex immunohistochemistry.

To further validate this result, we conducted experiments in mice. Four groups were established: the control group (G1), COG 133 TFA (anti‐APOE) treatment group (G2), anti‐programmed cell death protein 1 (PD1) treatment group (G3), and COG 133 TFA + anti‐PD1 dual treatment group (G4). The experimental schedule is shown in **Figure** **6**a. Starting from day 8, we conducted in vivo imaging to observe the changes in tumor size in each group of mice. As shown in Figure 6b, there are mice deaths both in the PBS group and the PD‐1 group. Compared to the tumor baseline before treatment, the COG 133 TFA + anti‐PD1 dual treatment group exhibited the highest tumor suppression effect, indicating a statistically significant difference (Figure 6c). We further dissected the tumors and measured their volume and weight. The combination of COG 133 TFA and anti‐PD1 dual treatment can produce significant tumor suppression (Figure 6d,e), suggesting that COG 133 TFA can enhance the efficacy of ICI treatments. Subsequently, four groups of mouse tumor specimens underwent Hematoxylin‐eosin (H&E), ki67 IHC, and mIHC staining to investigate tumor proliferation potential and the relationship between APOE^+^ macrophages and CD8^+^ Tex cells. The tumor tissues were identified through H&E staining, as shown in Figure S6a (Supporting Information). Besides, it was observed that the ki67 positive rate in the other three groups was lower compared to the PBS group, with the dual treatment group having the lowest rate (Figure S6b, Supporting Information). The mIHC was used to further confirm the distance between APOE^+^ macrophages and CD8^+^ Tex cells (Figure 6f‐i). The results showed that in the dual‐treatment group, the number of cells whose distance is no more than 100 µm is significantly lower than in other groups. Moreover, the number of cells within 50 µm is highest in the PBS group, indicating that there is an interaction between APOE^+^ macrophages and CD8^+^ Tex cells leading to the failure of immunotherapy, while APOE inhibitors can enhance the efficacy of immunotherapy (Figure 6j‐m).

## 3 Discussion

Recently, ICIs have gradually evolved into first‐line and new adjuvant treatments for most early‐stage and advanced cancers. Previous studies based on scRNA‐seq and transcriptomics methods have suggested a relationship between TAMs and CD8^+^ Tex cells in multiple cancers, and CD8^+^ T cells are preferentially located in the TAM‐rich region in the TME.^[^ ^22^ ^]^ This finding reveals that TAMs inhibit antitumor T‐cell immunity in solid tumors, which is related to poor prognosis and anticancer treatment failure.^[^ ^23^ ^]^ In this study, we analyzed the expression of genes associated with macrophages in ICI responders and non‐responders, and from eight machine learning techniques, we chose the svm algorithm to build an M.sig model that can precisely predict the response to ICIs in pan‐cancer. On this basis, we discovered that APOE^+^ macrophages, which are closely connected to CD8^+^ Tex cells, may be one of the causes of the failure in TNBC immunotherapy.

Previous studies have focused on exploring the relationship between macrophages and clinical characteristics. Long et al. established a macrophage‐related signature that can predict prognosis by using breast cancer RNA‐seq data, and its corresponding risk score is closely related to the pathological features of tumors and targeted therapy IC50s.^[^ ^24^ ^]^ Recently, the Nixon research team obtained a TAM‐related gene signature and confirmed that it may be related to cytotoxic T lymphocyte (CTL) depletion in a variety of malignant tumors but did not explain its role in immunotherapy.^[^ ^18^ ^]^ In addition, many researchers have found that macrophage‐related genes or gene signatures have an impact on TME and immunotherapy.^[^ ^25^ ^]^ In recent years, applying machine learning in medicine for prediction has gradually become a trend, including survival, metastasis, and treatment effects, with good results.^[^ ^26^ ^]^ However, this approach has not been applied to macrophages, especially for predicting response to immunotherapy. In this study, we built an ICI response prediction model using various machine learning algorithms and comprehensive scRNA‐seq and bulk transcriptome data, improving the accuracy of prediction and demonstrating innovation.

Compared with other gene signatures that predict the efficacy of immunotherapy, the AUC of the M.Sig model was higher in both the total cohort and separate cohorts, indicating that the model established has wide applicability among cancer types. Most of the M.Sig model genes are not included in the above 13 immune gene signatures.^[^ ^25^, ^27^ ^]^ MRC1 is considered a marker of M2 macrophages, and lapatinib (tyrosine kinase inhibitor) can downregulate its expression, thereby eliminating the invasion and migration of cancer cells mediated by M2 polarized macrophages.^[^ ^28^ ^]^ To explore the potential mechanism by which the M.Sig model can predict immunotherapeutic response, we divided macrophages into eleven subgroups and compared differences between them and their relationship with the M.Sig model. Among them, SPP1^+^ macrophages are a common subset that participates in tumor angiogenesis through interaction with SPP1‐CD44 of adjacent cancer‐associated fibroblasts (CAFs); these cells are related to the epithelial‐mesenchymal transformation (EMT) and poor prognosis of cancer patients.^[^ ^29^ ^]^ Additionally, APOE^+^ macrophages, CTSB^+^ macrophages, FOLR2^+^ macrophages, and S100A9^+^ macrophages accounted for the largest proportion of non‐responders, with the first three having the highest M.Sig.Score. This indicates that these macrophages may have the greatest suppressive impact on immunotherapy. It has been verified in a previous study on hepatocellular carcinoma,^[^ ^30^ ^]^ revealing that FOLR2^+^ macrophages express high levels of immunomodulatory chemokines (such as CXCL12 and CXCL16) and are located around the blood vessels of tumor immune privileged sites; thus, they play a major role in immunosuppressive interactions. Recently, Cao et al. found that CTSB in macrophages and the TME mature under the mediation of O‐GlcNAc transfer (OGT), promoting cancer metastasis and chemotherapy resistance, which is related to poor prognosis and consistent with the conclusions of this study.^[^ ^31^ ^]^

We analyzed the corresponding M.Sig.Score in order to further understand the function of macrophage subsets, and the results revealed that in TNBC, the difference in score of APOE^+^ macrophages between responders and non‐responders was particularly notable. Therefore, it is hypothesized that APOE^+^ macrophages may play a significant role in the immunotherapy's failure. Numerous studies have demonstrated that macrophage subsets can influence T cell activity and the immune microenvironment's ability to fight tumors. Additionally, our findings demonstrated the connection between APOE^+^ macrophages and CD8^+^ Tex cells and supported it with in vivo mice models and mIHC. Although Tang et al.^[^ ^32^ ^]^ have confirmed that APOE inhibitors combined with ICIs have anti‐tumor effects, our study further demonstrated that the combination can decrease M2 and CD8^+^ Tex cells in TNBC, which may be a crucial mechanism for enhancing the response of ICIs. Extracellular vesicles (EV) produced by APOE‐deficient animals can enhance the gene expression of inflammatory cytokines and M1 macrophage markers, while decreasing the gene expression of M2 macrophage markers, stimulating the growth of CD4^+^ T cells and triggering the production of IFN‐*γ* in T cells.^[^ ^33^ ^]^ It has been previously reported that the spatial distance between immune cells in the tumor microenvironment affects patient biological events. Biagio et al. found that the distance between tumor cells and CD8^+^ PD‐1^+^ T cells in non‐small cell lung cancer tissue increased after immunotherapy.^[^ ^34^ ^]^ Moreover, the distance between CD8^+^ Treg and tumor cells in lung cancer tissue is related to the prognosis of patients, specifically, the longer the distance, the better the prognosis of patients.^[^ ^35^ ^]^ In a study of TNBC, the patient's pathological complete response (pCR) is associated with the reduction of distance between tumor cells and CD3^+^ cells, as well as CD3^+^ CD8^+^ cells.^[^ ^36^ ^]^ These findings show the potential validity of our hypotheses and provide a theoretical framework for improving the efficacy of ICI‐based immunotherapy in the future.

However, this study has some limitations. First, the scRNA‐seq data used to construct the M.Sig model contained only five cancer types, resulting in a certain bias. The role of this model needs to be further verified in more kinds of cancers. Second, the results of this study were based on large‐scale public data from the real world, and biological interpretation requires further experiments in vitro to determine the credibility of the conclusions.

## 4 Conclusion 

In this study, we for the first time used large‐scale pan‐cancer scRNA‐seq and bulk RNA‐seq data from immunotherapy cohorts to develop an M.Sig model for ICI response prediction and stratification of prognosis with machine learning algorithms. In addition, we preliminarily explained the potential mechanism in TNBC by which the M.Sig model can predict the effect of ICI therapy, namely, by APOE^+^ macrophages interacting with CD8^+^ Tex cells. Our study provides guidance and ideas for resolving the heterogeneity of ICI response.

## 5 Experimental Section

### ICI scRNA‐seq Cohorts

Six immunotherapy cohorts were collected with both ICI response and scRNA‐seq data to investigate the relationship between macrophages and ICI efficacy. The data for these cohorts were available through GEO, Single Cell Portal (SCP), and Sequence Read Archive (SRA), as follows: SKCM_GSE120575,^[^ ^37^ ^]^ SKCM_GSE115978,^[^ ^27k^ ^]^ RCC_SCP1288,^[^ ^23b^ ^]^ TNBC_GSE169246,^[^ ^38^ ^]^ and BCC_GSE123813.^[^ ^39^ ^]^ Patients with partial response (PR) or complete response (CR) were classified as responders; those with progressive disease (PD) or stable disease (SD) were classified as non‐responders. The “Seurat” R package^[^ ^40^ ^]^ was utilized to analyze the cohorts. All cell annotation was accomplished, and macrophage data were extracted from each cohort. The data using the algorithm for canonical correlation analysis (CCA) was incorporated. Using the “FindMarkers” program and the criteria log2FC>0 and adjusted p value 0.05, variously expressed genes were identified, and differential gene analysis between responders and non‐responders was performed. Then, the “monocle3” R package^[^ ^41^ ^]^ was used to examine the cell development trajectory to display the responder and non‐responder distribution.

### ICI RNA‐seq Cohorts

Besides the scRNA‐seq cohorts, transcriptome data and clinical information for samples from 11 ICI RNA‐seq cohorts, including two BLCA cohorts (Imvigor210,^[^ ^42^ ^]^ GSE111636), two SKCM cohorts (GSE78220,^[^ ^27j^ ^]^ PRJEB23709^[^ ^43^ ^]^), one STAD cohort (PRJEB25780),^[^ ^44^ ^]^ one glioblastoma (GBM) cohort (PRJNA482620),^[^ ^45^ ^]^ one UC cohort (Zenodo546110),^[^ ^46^ ^]^ one NSCLC cohort (GSE126044),^[^ ^47^ ^]^ one BRCA cohort (GSE173839),^[^ ^48^ ^]^ one RCC cohort (GSE67501),^[^ ^49^ ^]^ and one MPM cohort (GSE99070)^[^ ^50^ ^]^ were systematically collected. The “clusterProfiler” R package was applied to convert ensemble IDs to gene symbols,^[^ ^51^ ^]^ and the data mentioned above were integrated with the “sva” R package.^[^ ^52^ ^]^ Probes were mapped using the “AnnoProbe” R package (https://github.com/jmzeng1314/AnnoProbe), and the “limma” R package^[^ ^53^ ^]^ was used to calculate the average values of multiple probes if necessary.

### Establishment of M.Sig Model and M.Sig.Score

The integrated ICI RNA‑Seq dataset was randomly divided into training and validation cohorts at a ratio of 8:2. Model training and validation with the “mlr3” R package^[^ ^54^ ^]^ with the differential genes above were accomplished. Eight machine learning algorithms, including svm, randomForest, k‐nearest neighbors (knn), naïve bayes (nb), cross‐validation glmnet (cv_glmnet), decisionTree, extreme gradient boosting (xgBoost), and logistic regression (lr), were used for the training cohort. Each method was evaluated using tenfold CV, and the effectiveness of the models were determined by calculating the AUC. To meet the criteria of a better AUC value and fewer variables, the “mlr_filters” function with the parameter “jmim” was used to calculate importance scores for each gene and reduced the features from lowest to highest priority ratings.

To further assess the effectiveness of the M.Sig model, thirteen ICI response signatures were gathered (IMS.Sig,^[^ ^27a^ ^]^ ImmmunCells.Sig,^[^ ^25c^ ^]^ INFG.Sig,^[^ ^27b^ ^]^ T.cell.infamed.Sig,^[^ ^27b^ ^]^ PDL1.Sig,^[^ ^27c^ ^]^ TcellExc.Sig,^[^ ^27k^ ^]^ CRMA.Sig,^[^ ^27d^ ^]^ NLRP3.Sig,^[^ ^27e^ ^]^ Cytotoxic.Sig,^[^ ^27f^ ^]^ TRS.Sig,^[^ ^27g^ ^]^ LRRC15.CAF.Sig,^[^ ^27h^ ^]^ IMPRES.Sig,^[^ ^27i^ ^]^ and IPRES.Sig^[^ ^27j^ ^]^). Codes and algorithms for these signatures were derived from the original studies.

This study examined various levels of expression for each gene of the M.Sig model in the ICI RNA‐Seq data according to patient response. Using the Single‐sample Gene Set Enrichment Analysis (ssGSEA) algorithm, it screened up‐regulated and down‐regulated genes in non‐responders and calculated M.Sig.Score using the following formula:

(1) $$\begin{array}{ccc} M.Sig.Score & = & ssGSEA.Score\;\left(up-regulated\right)\\ & & -\;ssGSEA.Score\;\left(down-regulated\right) \end{array}$$

### Bulk Transcriptomic Data

Transcriptomic data was downloaded from the pan‐cancer cohort of TCGA from the UCSC Xena data portal (https://xenabrowser.net). Classic immunomodulators and TME scores determined by “MCPcounter” algorithms^[^ ^55^ ^]^ were obtained from a previously published article.^[^ ^56^ ^]^ Additionally, TMB data were acquired to examine the link between the M.Sig model and the TMB. Then, MSI data from colon adenocarcinoma (COAD) patients were collected to determine a distinction between MSI statuses. Data of TNBC patients were downloaded from the GEO database, including GSE114269,^[^ ^57^ ^]^ GSE21653^[^ ^58^ ^]^ and GSE173839. All the datasets was used is summarized in Table S1 (Supporting Information).

### Functional Enrichment Analysis

Enrichment analysis based on the KEGG and GO databases was performed using the “clusterProfiler” R package.^[^ ^51^ ^]^ The ssGSEA was conducted on each sample with the “GSVA” R package,^[^ ^59^ ^]^ which was used to calculate the “Exhausted score” with the corresponding gene set and “APOE score” with the top20 feature genes (Table S2, Supporting Information).

### Cell–Cell Interaction (CCI) Analysis

The R package “CellChat”^[^ ^60^ ^]^ identifies differentially expressed signaling genes and calculates the collective average expression, then uses the law of mass action to model ligand‐receptor mediated signaling interactions and adopts a random walk‐based network propagation technique. Calculate inter‐cell communication probabilities and ultimately determine statistically significant inter‐cell communication. So, it applied it to explore cell interactions and determine the mechanism of communication molecules at the single‐cell level.

The R package “SingleCellSignalR”^[^ ^61^ ^]^ utilizes a manually curated database and calculates probabilities as a linear function of the product of ligand and receptor expression. Then it used the package for systematic analysis of ligand and target gene pairs. The gene expression data of interacting cells was input into SingleCellSignalR and combined with a prior model that integrates existing knowledge of ligand target signaling pathways. Then, predict the ligand receptor interactions that drive changes in gene expression in cells of interest.

### mIHC Staining of TNBC Tissue

Human and mouse TNBC tissues were fixed with 4% paraformaldehyde before being embedded in paraffin. Tissue sections (4 µm) were baked in an oven at 65 °C for 1 h to improve sample adhesion to the slide. Then, they were dewaxed with fresh xylene twice for 15 min each and rehydrated with graded alcohol (100%, 95%, 85%, 75%). The samples were then transferred to the proper antigen retrieval (AR) solution after being fixed for 20 min at room temperature in 10% neutral‐buffered formalin (NBF) (Solarbio, #G2161, China). The slices were then microwaved for 15 min at 20% power after 1 min at 100% power. After being blocked and brought to room temperature, the slides were incubated with the primary antibody for 10 min. Slides were washed three times with TBST (Solarbio, #T1082, China) to get rid of any extra antibodies. Slides were then exposed to Opal Polymer HRP Ms+Rb (AKOYA Biosciences, #NEL820001KT, USA) for 10 min at room temperature. Slides were rinsed three times with TBST to remove any remaining wash buffer before being incubated with Opal Signal Generation. Before each additional antibody incubation, the steps of microwave treatment, blocking, primary antibody incubation, and introduction of Opal Polymer HRP were carried out once again. After all the primary antibodies were done, the slides were then incubated with DAPI working solution for 5 min in the dark at room temperature. After that, slides were mounted after being cleaned with distilled water and TBST. Finally, photographs of those tissue samples were taken using a confocal microscope (Nikon, Japan) or a Vectra Polaris Quantitative Pathology Imaging System. Intercellular distance measurements of mouse tissues were performed with HALO software. In the human TNBC samples, ImageJ (version 1.54 g) was used with Java 8.0 (64‐bit) to record the total number of cells and the centroid positions of each cell. Then, functions from the OpenCV (version 4.7.0) library were utilized to compare the pixel color values of each cell's centroid position in Python software. For cells expressing common colors, libraries such as NumPy (version 1.24.4) and Pandas (version 2.1.1) were employed to calculate distances between cells.

Antibodies used in human tissues include CD68 (1:1000, #ab213363, Abcam), CD8a (1:400, #66868‐1‐Ig, Proteintech), PD‐1 (1:400, #66220‐1‐Ig, Proteintech), APOE (1:100, #66830‐1‐Ig, Proteintech), and IFNγ (1:100, #15365‐1‐AP, Proteintech). And in mouse tissues, CD8 (1:200, #ab217344, Abcam), PD‐1 (1:4000, #66220‐1‐Ig, Proteintech), F4/80 (1:8000, #28463‐1‐AP, Proteintech), and APOE (1:200, #A0304, Abclonal) were used.

### Mice Model

Female BALB/c mice (6 weeks old, immunocompetent) were purchased from Zhejiang Center of Laboratory Animals (ZJCLA) and housed in the ZJCLA. All animal husbandry and experimental procedures, including animal housing and diet, were performed under the guidelines, and were approved by the Institutional Animal Care and Use Committee (IACUC) and ZJCLA (ethical number: ZJCLA‐IACUC‐20010267). The 4T1 cell line was purchased from FuHeng BioLogy cultured with RPMI 1640 medium (Meilun Biotechnology, #MA0215‐2, China) supplemented by 10% fetal bovine serum (FBS) (Cellmax, #SA211.02, USA) at 37 °C in a 5% CO^2^ chamber. The firefly luciferase gene, which was added via lentiviral transduction, was expressed consistently by the 4T1‐Luc cell line. For animal model, fifty microlitres of 2.5 × 10^5^ 4T1‐Luc cells mixed with Matrigel (Yeasen, #40185ES10, China) were inoculated into the fourth mammary fat pad of mice to establish the subcutaneous tumor‐bearing model. One week later, all tumor‐bearing mice were divided randomly into four groups (n = 11). From the 8th day, COG133TFA (αAPOE, MCE, #HY‐P1050A, USA) (1 mg kg^−1^, i.p.) dissolved in PBS (100 µL) were conducted every 3 days for a total of four times. Treatments with anti‐PD‐1 (5 mg kg^−1^, i.p.) were conducted every 4 days for four times. Among these mice, 24 were used for in vivo imaging experiments with D‐Luciferin, Potassium Salt D (Yeasen, #40902ES03, China) to test drug efficacy, and 20 for tumor volume and weight measurements. Tumor volumes were calculated according to the modified ellipsoidal formula: V = 1/2 (length × width × width). (Figure 6A). All mice were sacrificed and tumor tissues were collected for H&E, ki67 IHC and mIHC staining, and the methods of H&E and ki67 IHC staining were elaborated in our previous study.^[^ ^62^ ^]^

### Statistical Analysis

All statistical analyses were performed using R software (version 4.1.0). The Wilcoxon test was employed to analyze differences between two groups and the Kruskal–Wallis test to analyze differences between more than two groups. Survival curves, which are described by Kaplan–Meier plots, were compared with the log‐rank test. Wilcoxon test was carried out to see the statistical difference between two groups. Correlation coefficients were calculated as Spearman correlations. *P* < 0.05 was considered statistically significant. Significant *P*‐values were denoted as follows: 0 ≤ ****<0.0001≤***< 0.001 ≤** < 0.01 ≤* < 0.05.

## Conflict of interest

The authors declare no conflict of interest.

## Author Contributions

C.L., J.X., B.L., and W.T. contributed equally to this work. C.L. performed methodology, formal analysis, visualization, and wrote the original draft. J.X. performed methodology, formal analysis, visualization, and wrote the original draft. B.L. performed methodology, software, validation. W.T. performed formal analysis, validation, wrote the original draft, and reviewed and edited the final manuscript. Y.W. performed formal analysis, methodology, software. S.X. performed data curation and validation. L.H. performed data curation and validation. X.L. performed data curation and validation. L.L. performed data curation and validation. Y.J. performed data curation and validation. H.T. performed data curation and validation. X.D. performed data curation and validation. Y.Z. performed data curation and validation. S.Z. performed data curation and validation. W.F. performed data curation and validation. J.C. performed software, supervision, wrote the original draft and reviewed and edited the final manuscript. X.D. performed conceptualization, supervision, funding acquisition, and data curation. X.B. performed supervision, funding acquisition, conceptualization, and project administration. P.Z. performed supervision, funding acquisition, conceptualization, project administration, wrote the original draft and reviewed and edited the final manuscript.

## Supporting information

Supporting Information

Supplemental Table 2

## Data Availability

Data sharing is not applicable to this article as no new data were created or analyzed in this study.
