# Supplementary material for: Pan‐Cancer Single‐Cell and Spatial‐Resolved Profiling Reveals the Immunosuppressive Role of APOE+ Macrophages in Immune Checkpoint Inhibitor Therapy
Source: Adv Sci (Weinh). 2024 Apr 3;11(23):2401061. doi: 10.1002/advs.202401061 (PMC11186051; doi:10.1002/advs.202401061)
Supplement: Supplementary file 1 — Supporting Information [file ADVS-11-2401061-s002.pdf]

ADVANCED SCIENCE
Open Access

Supporting Information

for *Adv. Sci.*, DOI 10.1002/advs.202401061

Pan-Cancer Single-Cell and Spatial-Resolved Profiling Reveals the Immunosuppressive Role of APOE+ Macrophages in Immune Checkpoint Inhibitor Therapy

Chuan Liu, Jindong Xie, Bo Lin, Weihong Tian, Yifan Wu, Shan Xin, Libing Hong, Xin Li, Lulu Liu, Yuzhi Jin, Hailin Tang, Xinpei Deng, Yutian Zou, Shaoquan Zheng, Weijia Fang, Jinlin Cheng, Xiaomeng Dai*, Xuanwen Bao* and Peng Zhao**

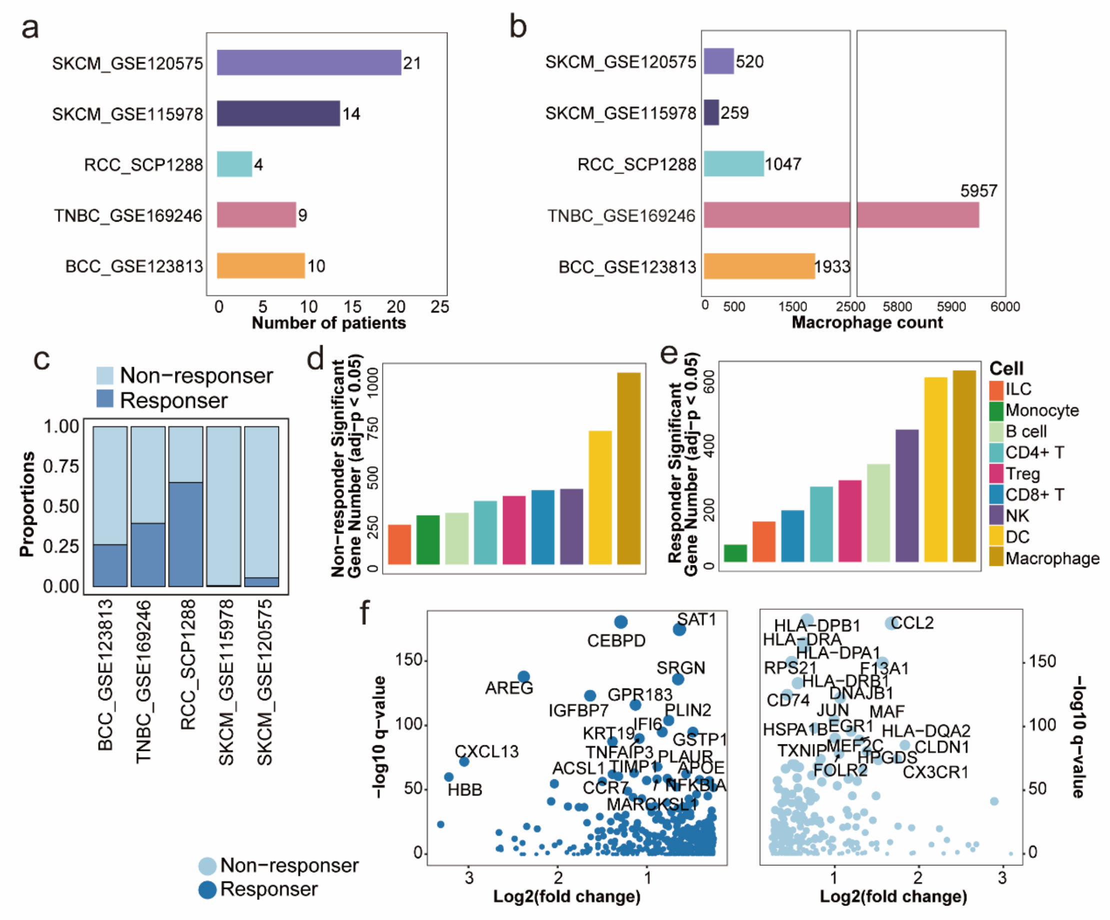

Figure S1: (a) Numbers of patients in five cohorts. (b) Numbers of macrophages in five cohorts. (c) Different proportions of ICI outcomes in five cohorts. (d) Number of DEGs of each cell type in responders. (e) Number of differential genes of each cell type in non-responders. (f) Distinction expression profiles of macrophages between responders and non-responders. ICI: immune checkpoint inhibitor; DEGs: differential expressed genes.

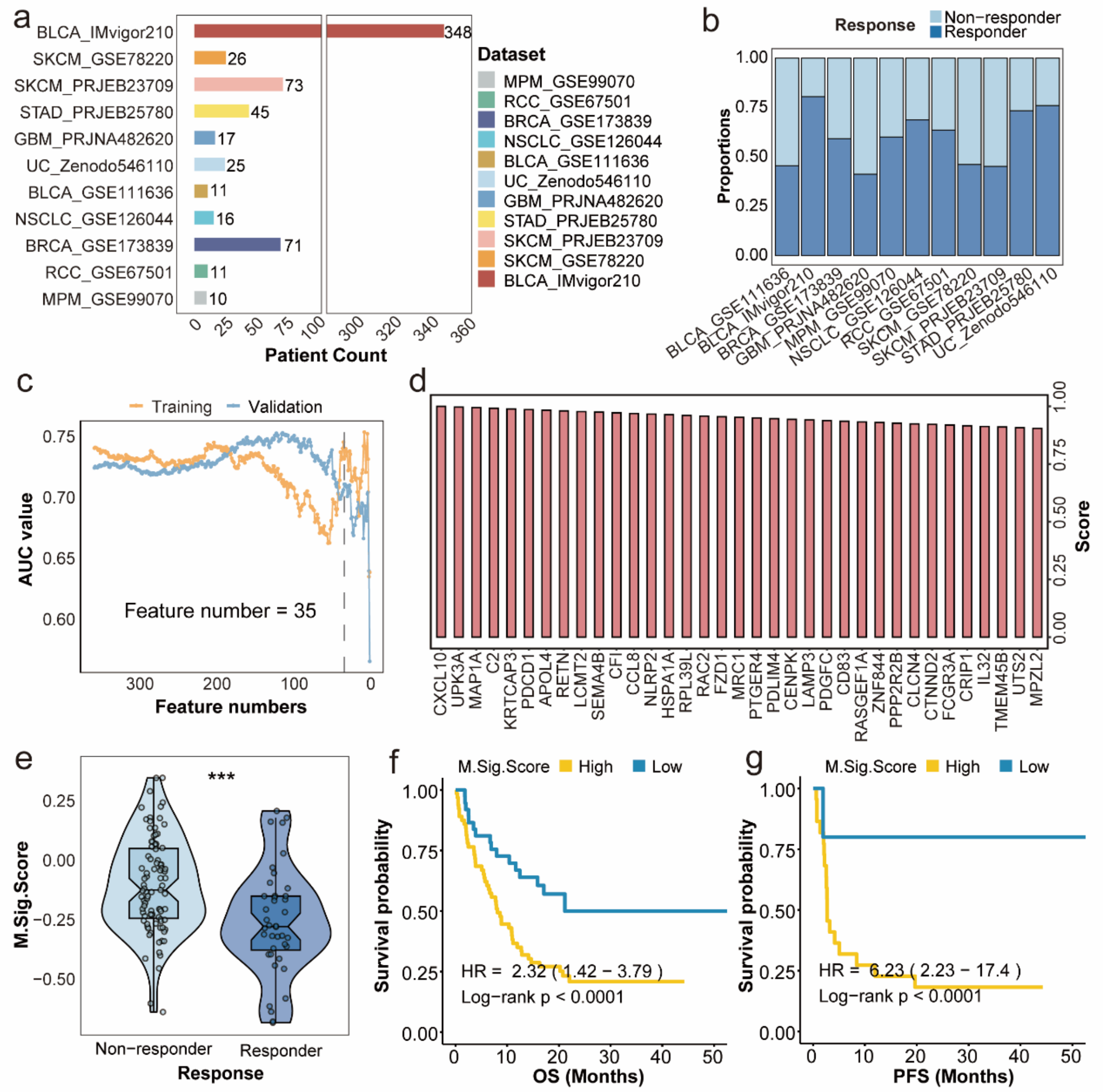

Figure S2: (a) Number of patients in each dataset. (b) Different proportions of ICI outcomes in each dataset. (c) The relationship between feature numbers and AUC values features of the svm model. (d) The 35 features were included as the M.Sig model. (e) Different M.Sig.Scores between responders and non-responders in the verification set. (f-g) OS and PFS between high and low M.Sig.Scores in the verification set. ICI: immune checkpoint inhibitor; AUC: area under the curve; OS: overall survival; PFS: progression-free survival; svm: support vector machine.

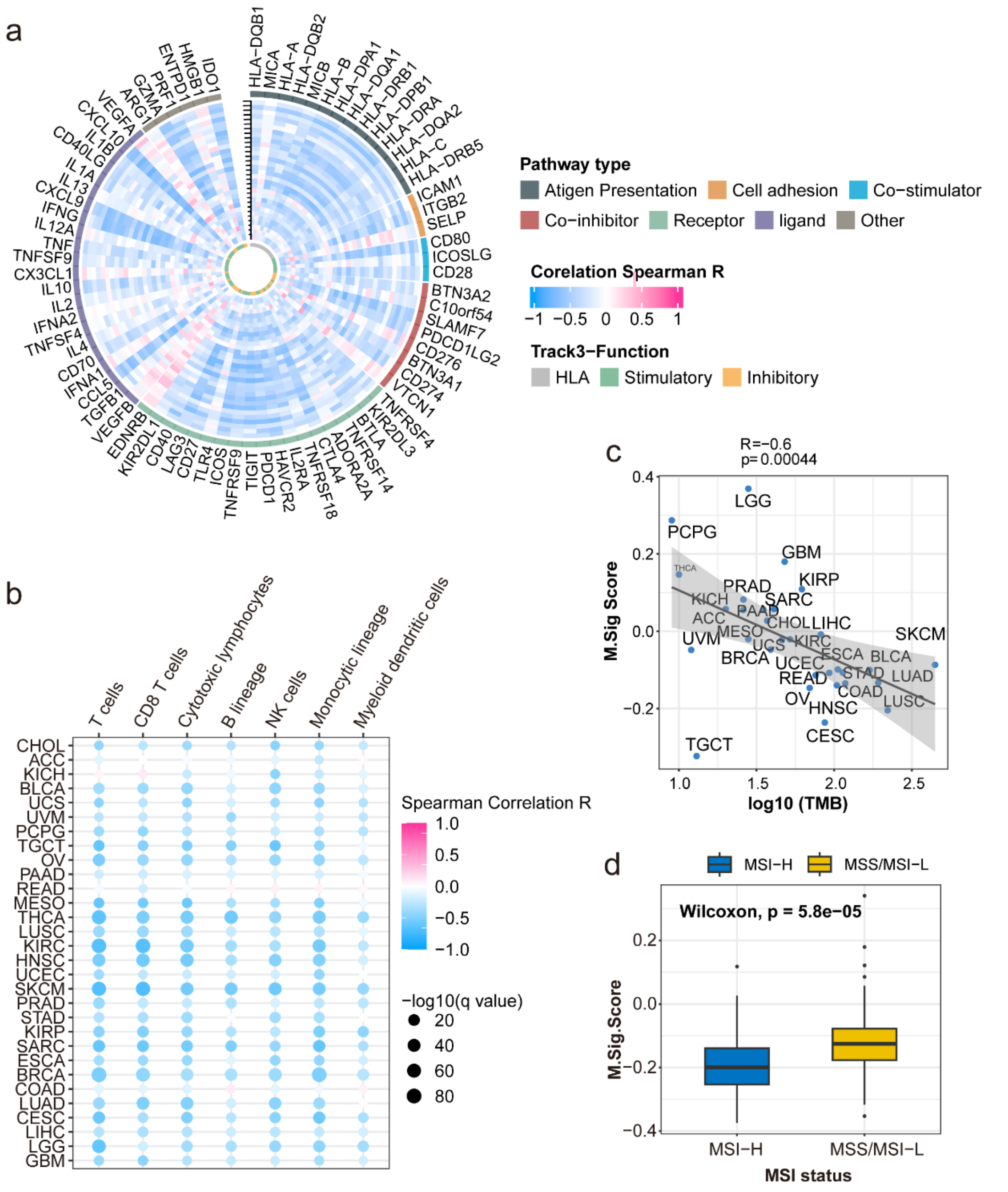

Figure S3: (a) The association between M.Sig.Score and immune-related genes and cell populations. (b) The cell abundance scores were calculated with the gene set using the ssGSEA method on the TCGA bulk transcriptome data. (c) The association between the M.Sig model and TMB in TCGA pan-cancer cohort. (d) The association between the M.Sig model and MSI status in TCGA pan-cancer cohort. ssGSEA: Single-sample Gene Set Enrichment Analysis; TCGA: The Cancer Genome Atlas; TMB: tumour mutation burden; MSI: microsatellite instability.

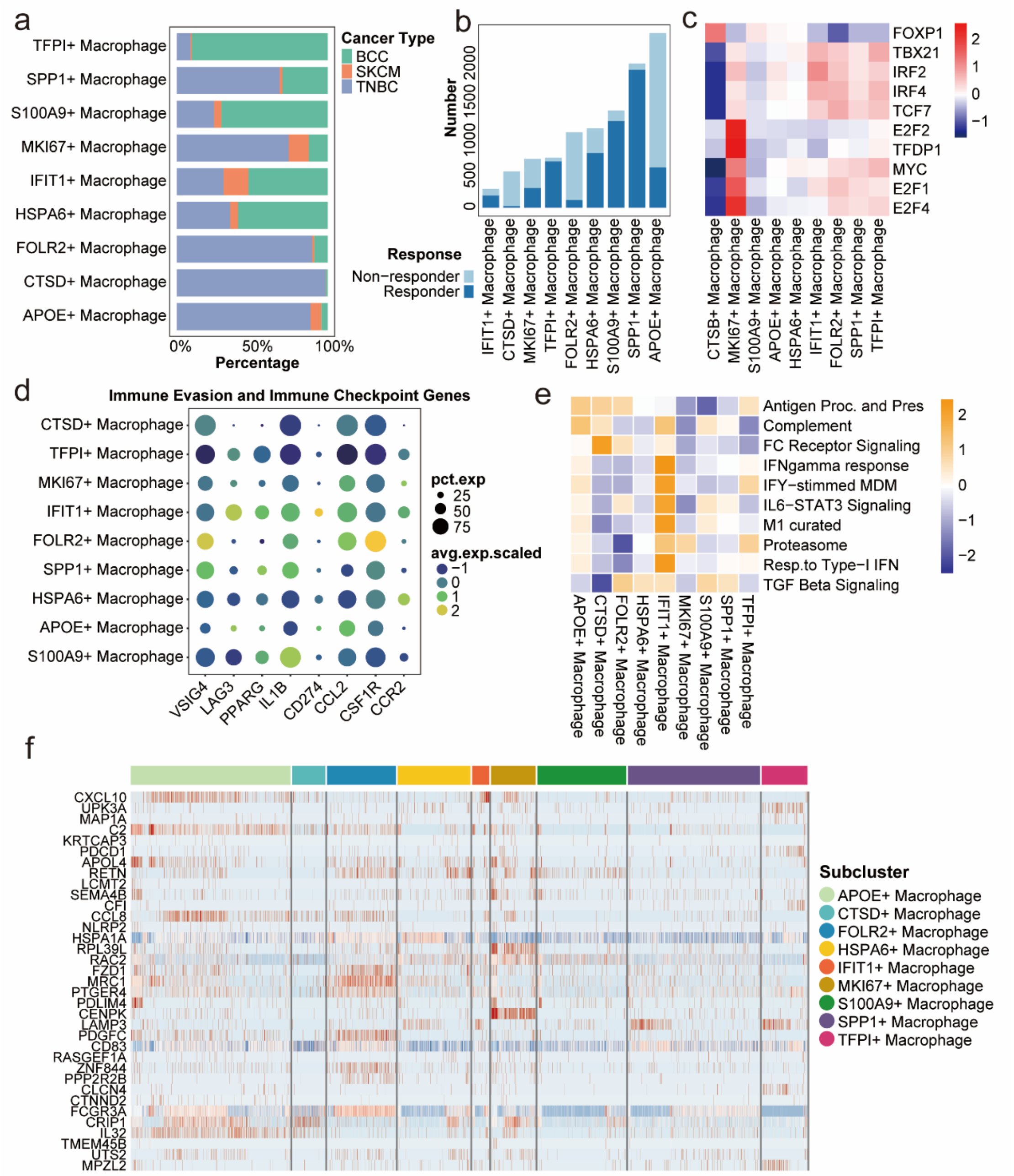

Figure S4: (a) The proportion of cancer types in macrophage subpopulations. (b) Number of responders and non-responders in macrophage subgroups. (c-e) Comparison of transcription factor activities, immune evasion and immune checkpoint genes, and immune-related pathways among macrophage subclusters. (f) Expression of 35 genes from the M.Sig model in macrophage subpopulations.

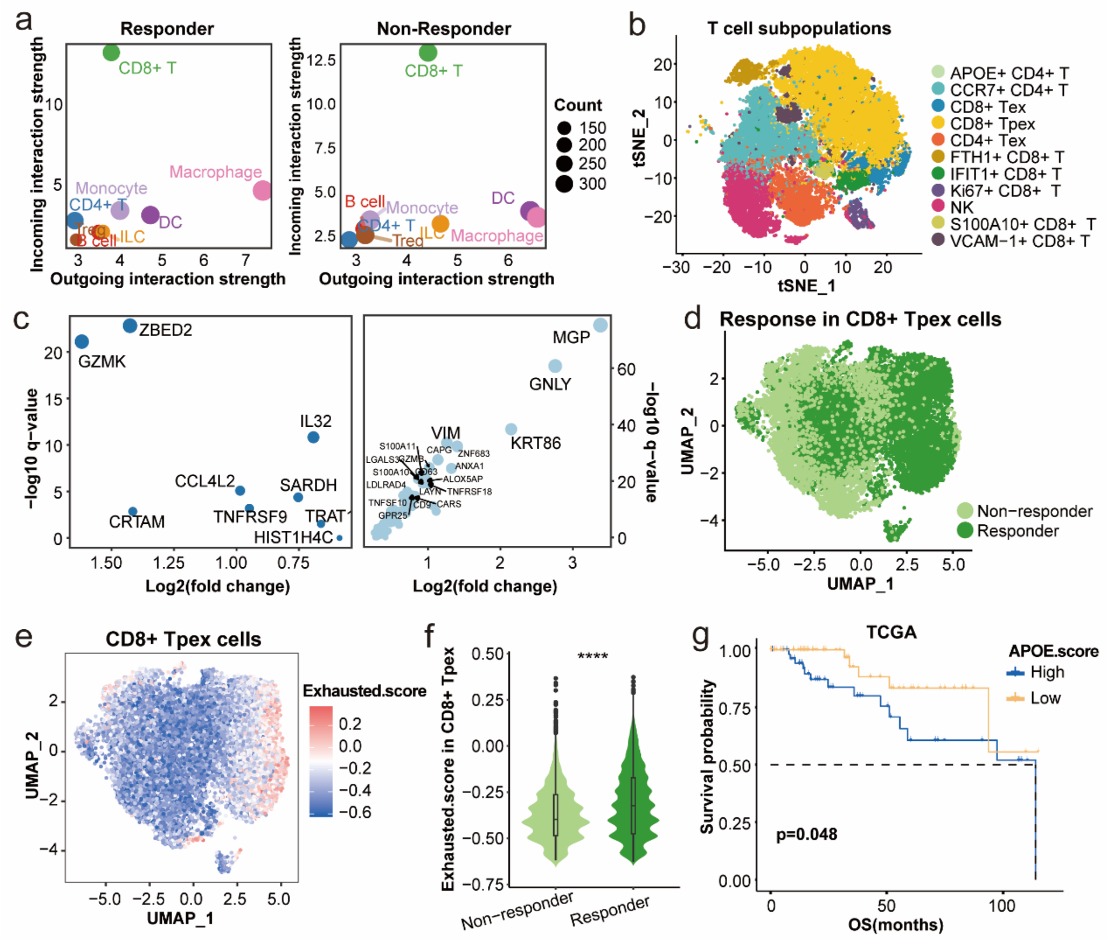

Figure S5: (a) The relationship between differential outgoing interactions and incoming interaction strength for nine cell types in responders and non-responders. (b) Colour-coded tSNE plot of T cell subgroups. (c) Distinction expression profiles of T cells between responders and non-responders. (d) Colour-coded UMAP plot of responders and non-responders in CD8^{+} Tpex cells. (e) Exhausted.score of CD8^{+} Tpex cells in UMAP. (f) Comparison of Exhausted.score in CD8^{+} Tpex cells between responders and non-responders. (g) OS between high and low APOE.score in TNBC from TCGA cohort. tSNE: t-distributed stochastic neighbour embedding; UMAP: uniform Manifold Approximation and Projection; Tpex cells: pro-exhausted T cells; OS: overall survival; TNBC: triple negative breast cancer; TCGA: The Cancer Genome Atlas.

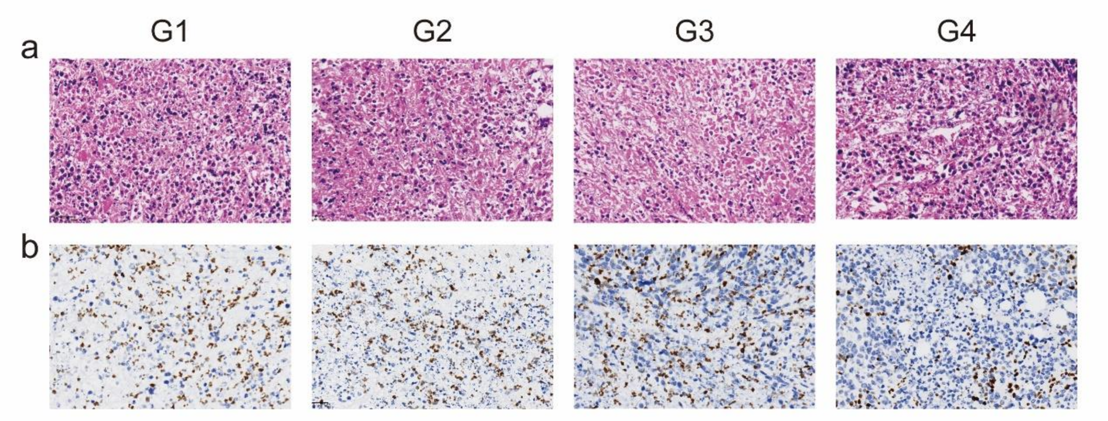

Figure S6: (a) H&E staining of mice breast cancer sections from G1-G4. (b) Ki67 immunohistochemistry of mice breast cancer sections from G1-G4. H&E: Hematoxylin-eosin.

Table S1 List of all datasets used in the study.

Dataset	Data type	Cancer type	All Patients	Treatment
GSE120575	scRNA-seq	SKCM	21	aPD-1, aCTLA4, Combo
GSE115978	scRNA-seq	SKCM	14	aPD-1
GSE169246	scRNA-seq	TNBC	9	aPD-L1
GSE123813	scRNA-seq	BCC	10	aPD-1
SCP1288	scRNA-seq	RCC	4	aPD-1, aPD-1+aCTLA4
GSE111636	Bulk RNA-seq	BLCA	11	aPD-1
Imvigor210	Bulk RNA-seq	BLCA	348	aPD-L1
GSE78220	Bulk RNA-seq	SKCM	26	aPD-1
PRJEB23709	Bulk RNA-seq	SKCM	73	aPD-1
PRJEB25780	Bulk RNA-seq	STAD	45	aPD-1
PRJNA482620	Bulk RNA-seq	GBM	17	aPD-1
Zenodo546110	Bulk RNA-seq	UC	25	aPD-L1
GSE126044	Bulk RNA-seq	NSCLC	16	aPD-1
GSE67501	Bulk RNA-seq	RCC	11	aPD-1
GSE99070	Bulk RNA-seq	MPM	10	aPD-1
GSE173839	Bulk RNA-seq	BRCA	71	aPD-L1
GSE114269	Bulk RNA-seq	BRCA	53	NA
GSE21653	Bulk RNA-seq	BRCA	85	NA
TCGA	Bulk RNA-seq	Pan-cancer	10228	NA
